# Supplementary material for: Native Microbes Amplify Native Seedling Establishment and Diversity While Inhibiting a Non-Native Grass
Source: Plants (Basel). 2023 Mar 6;12(5):1184. doi: 10.3390/plants12051184 (PMC10005557; doi:10.3390/plants12051184)
Supplement: Supplementary file 1 [file plants-12-01184-s001.zip › Supplement S1. Figures Tables.pdf]

Supplement S1. Koziol et. al.

Supplemental results on weed recruitment.

Table S1. Seed list

Table S2. Full intrinsic species list.

Table S3. First Harvest Results

Table S4. Second Harvest Results

Figure S1. Photograph of the pots prior to harvest.

Figure S2. The effect of inoculation across first and second harvests where the main effect of inocula was consistent between harvests.

Figure S3. The effect of inoculation across first and second harvests where the main effect of inocula increased or decreased between harvests.

Non-seeded plant recruitment

Several species were observed that were not seeded into the experiment. Our best guess is that these species were recruited in the ex-arable soil inocula or as weeds in the native seed mixture. *Amaranthus sp., Daucus carota, Bromus tectorum, Melilotus officinalis, Panicum oligosanthes, Pplyganum sp., Primula vulgaris, Euphorbia sp, Viola sororia, Panicum capillare, Andropogon viriginicus, Trifolium pratense* and *Medicago lupulina*. These species were included with the seeded non-native when comparing native ns non-native abundances. On average, the combined total biomass of these non-seeded weed species was 3% of entire pot biomass.

Table S1. Seed list with each plant's common and scientific name, functional group, coefficient of conservation (CC) score[1], seed source location, and whether it was observed in the study.

Common Name	Scientific Name	Functional Group	CC score	Seed Source	Observed in Study
Leadplant	*Amorpha canescens*	*Legume*	*7*	Hamilton	Yes
Milkweed, Swamp	*Asclepias incarnata*	*Forb*	*4*	Hamilton	Yes
Milkweed, Common	*Asclepias syriaca*	*Forb*	*1*	Hamilton	Yes
Milkweed, Butterfly	*Asclepias tuberosa*	*Forb*	*6*	Hamilton	Yes
Indigo, Blue	*Baptisia australis*	*Legume*	*6*	Hamilton	Yes
Sunflower, Tickseed	*Bidens aristosa*	*Forb*	*3*	Hamilton	Yes
Indian Paintbrush	*Castilleja coccinea*	*Forb*	*4*	Hamilton	No
Partridge Pea	*Chamaecrista fasciculata*	*Legume*	*2*	Hamilton	Yes
Coreopsis, Grand	*Coreopsis grandiflora*	*Forb*	*8*	Hamilton	No
Coreopsis, Tickseed	*Coreopsis lanceolata*	*Forb*	*7*	Hamilton	Yes
Coreopsis, Plains	*Coreopsis tinctoria*	*Forb*	*1*	Hamilton	Yes
White Prairie Clover	*Dalea candidum*	*Legume*	*7*	Prairie Moon	Yes
Purple Prairie Clover	*Dalea purpurea*	*Legume*	7	Prairie Moon	Yes
Illinois Bundleflower	*Desmanthus illinoensis*	*Legume*	*2*	Hamilton	Yes
Coneflower, Pale Purple	*Echinacea pallida*	*Forb*	*7*	Hamilton	Yes
Coneflower, Purple	*Echinacea purpurea*	*Forb*	*3*	Hamilton	Yes
Rattlesnake master	*Eryngium yuccifolium*	*Forb*	*7*	Hamilton	Yes
Foxtail	Setaria faberi	*Grass*	0	Field Collected	Yes
White Prairie Gentian	*Gentiana flavida*	*Forb*	8	Prairie Moon	No
Sunflower, Sawtooth	*Helianthus grosseserratus*	*Forb*	*4*	Hamilton	Yes
Sunflower, Ox-eye	*Heliopsis helianthoides*	*Forb*	*5*	Hamilton	Yes
Lespedeza, Slender	*Lespedeza virginica*	*Legume*	*5*	Hamilton	Yes
Little Bluestem Grass	Schizachyrium scoparium	Grass	5	Hamilton	Yes
Seed Box	*Ludwigia alternifolia*	*Forb*	*5*	Hamilton	Yes
Sensitive Brier	*Mimosa quadrivalvis*	*Forb*	*6*	Hamilton	Yes
Bergamot, Wild	*Monarda fistulosa*	*Forb*	*3*	Hamilton	Yes
Sampson's Snakeroot	*Orbexilum pedunculatum*	*Forb*	*9*	Hamilton	Yes
Beardtongue, White	*Penstemon digitalis*	*Forb*	*4*	Hamilton	Yes
Downy flox	*Phlox pilosa*	*Forb*	7	Prairie Moon	No
Virginia Lions Heart	*Physostegia virginiana*	*Legume*	7	Prairie Moon	No
Slender Mtn Mint	*Pycnanthemum tenuifolium*	*Forb*	*4*	Hamilton	No
Coneflower, Upright Prairie	*Ratibida columnifera*	*Forb*	*4*	Hamilton	Yes
Rose, Prairie	*Rosa carolina*	*Forb*	*5*	Hamilton	No
Black-eyed Susan, Common	*Rudbeckia hirta*	*Forb*	*2*	Hamilton	Yes
Early Figwort	*Scrophularia lanceolata*	*Forb*	5	Prairie Moon	Yes
Senna	*Senna marlandica*	Legume	3	Hamilton	Yes
Goldenrod, Gray	*Solidago nemoralis*	*Forb*	*2*	Hamilton	No
Goldenrod, Rigid	*Solidago rigida*	*Forb*	*3*	Hamilton	Yes
Prairie Dropseed	*Sporobolus heterolepis*	*Grass*	*8*	Hamilton	Yes
Ohio Spiderwort	Tradescantia ohiensis	*Forb*	2	Prairie Moon	Yes
Culver's root	*Veronicastrum virginicum*	*Forb*	*8*	Hamilton	Yes
Golden Alexanders	*Zizia aurea*	*Forb*	*5*	Hamilton	Yes

Table S2. Full intrinsic species output.

Species	NMDS1	NMDS2	p-value
B. aristosa	0.4978811	-0.741185	0.001
E. pallida	0.3758625	-0.475515	0.001
C. fasciculata	0.4120677	0.7460587	0.001
S. faberi	-0.77588	-0.083553	0.001
E. yuccifolium	0.430899	-0.373672	0.002
H. helianthoides	0.3823069	-0.329738	0.003
R. columnifera	0.2631774	-0.388539	0.004
M. lupulina	0.2941195	-0.378259	0.004
A. artemisiifolia	0.2044813	0.4260718	0.005
S. scoparium	0.4244076	0.2123334	0.009
P. aviculare	0.0028758	0.4169241	0.016
V. sororia	0.2141607	-0.380134	0.018
R. hirta	0.4068963	0.0594642	0.024
UF (Unknown Forb)	0.1385269	-0.362878	0.03
B. alba	0.323966	0.1972003	0.035
S. lanceolata	0.0683162	0.3843318	0.036
A. tuberosa	0.2083887	-0.310465	0.045
P. cuspidatum	-0.042617	0.3576915	0.048
A. gerardii	0.1858767	0.4012776	0.049
C. lanceolata	0.3055674	0.1741912	0.071
A. retroflexus	0.0682149	0.3365135	0.076
C. hirsuta	0.2094112	-0.284899	0.08
C. tinctoria	0.1679516	-0.278248	0.09
M. officinalis	0.1491952	-0.308403	0.097
E. purpurea	0.2626425	-0.170812	0.11
P. albidus	0.2947405	-0.139059	0.113
Z. azurea	0.2487457	-0.175102	0.126
L. virginica	0.2586647	-0.141857	0.153
T. ohiensis	-0.158657	-0.245985	0.158
D. illinoensis	0.133213	-0.261549	0.161
E. serpens	0.2763653	-0.153518	0.165
D. candidum	0.277384	-0.0743	0.179
V. virginicum	-0.244972	0.0091704	0.284
L. alternifolia	-0.001821	0.2364501	0.298
D. carota	0.0827233	-0.196776	0.375
S. heterolepis	0.1942285	0.0133795	0.451
D. tectorum	0.0236724	-0.179563	0.481
H. grosseserratus	0.1682253	-0.055843	0.542

G. squarrosa	0.0441921	-0.142147	0.594
D. oligosanthes	0.0065178	-0.150447	0.623
P. virgatum	-0.103626	-0.09859	0.648
P. capillare	-0.093565	0.1031263	0.661
O. biennis	0.0442894	-0.105957	0.703
A. canescens	0.0986364	-0.068576	0.748
A. syriaca	-0.104058	0.0481726	0.751
M. microphylla	-0.039983	0.1075092	0.759
S. marilandrica	0.0132233	0.1143179	0.768
S. rigida	0.0048935	-0.114755	0.774
M. fistulosa	0.0958099	0.0190283	0.827
D. purpureum	-0.049126	0.0461818	0.904
O. pedunculatum	0.0026497	0.0076343	0.998

**Species**	**NMDS1**	**NMDS2**	**p-value**
B. aristosa	0.4978811	-0.741185	0.001
E. pallida	0.3758625	-0.475515	0.001
C. fasciculata	0.4120677	0.7460587	0.001
S. faberi	-0.77588	-0.083553	0.001
E. yuccifolium	0.430899	-0.373672	0.002
H. helianthoides	0.3823069	-0.329738	0.003
R. columnifera	0.2631774	-0.388539	0.004
M. lupulina	0.2941195	-0.378259	0.004
A. artemisiifolia	0.2044813	0.4260718	0.005
S. scoparium	0.4244076	0.2123334	0.009
P. aviculare	0.0028758	0.4169241	0.016
V. sororia	0.2141607	-0.380134	0.018
R. hirta	0.4068963	0.0594642	0.024
UF (Unknown Forb)	0.1385269	-0.362878	0.03
B. alba	0.323966	0.1972003	0.035
S. lanceolata	0.0683162	0.3843318	0.036
A. tuberosa	0.2083887	-0.310465	0.045
P. cuspidatum	-0.042617	0.3576915	0.048
A. gerardii	0.1858767	0.4012776	0.049
C. lanceolata	0.3055674	0.1741912	0.071
A. retroflexus	0.0682149	0.3365135	0.076
C. hirsuta	0.2094112	-0.284899	0.08
C. tinctoria	0.1679516	-0.278248	0.09
M. officinalis	0.1491952	-0.308403	0.097
E. purpurea	0.2626425	-0.170812	0.11
P. albidus	0.2947405	-0.139059	0.113
Z. azurea	0.248745682	-0.175101882	0.126
L. virginica	0.258664675	-0.141856715	0.153
T. ohiensis	-0.158656923	-0.245984531	0.158
D. illinoensis	0.133212985	-0.261548602	0.161
E. serpens	0.276365312	-0.153517549	0.165
D. candidum	0.277384022	-0.074299782	0.179
V. virginicum	-0.244972074	0.009170384	0.284
L. alternifolia	-0.001820594	0.236450071	0.298
D. carota	0.082723255	-0.19677555	0.375
S. heterolepis	0.19422846	0.013379474	0.451
D. tectorum	0.023672411	-0.179563342	0.481
H. grosseserratus	0.168225303	-0.05584329	0.542
G. squarrosa	0.044192096	-0.142147174	0.594
D. oligosanthes	0.006517825	-0.15044677	0.623
P. virgatum	-0.103626242	-0.098590004	0.648
P. capillare	-0.09356524	0.103126322	0.661
O. biennis	0.044289418	-0.10595681	0.703
A. canescens	0.098636362	-0.06857567	0.748
A. syriaca	-0.104058351	0.0481726	0.751
M. microphylla	-0.039982544	0.107509211	0.759
S. marilandrica	0.013223296	0.114317863	0.768
S. rigida	0.004893479	-0.1147546	0.774
M. fistulosa	0.095809866	0.01902825	0.827
D. purpureum	-0.049126286	0.046181751	0.904
O. pedunculatum	0.002649747	0.007634337	0.998

Table S3. The main effect of inoculation (bold) and contrasts results from our PROC GLM model in SAS on total abundance and by nativeness and successional stage (A) and for plant community diversity and by functional group (B) for the first harvest.

Table S3A. Harvest 1		**Total Diversity**		**Total Abundance**		**Native Abundance**		**Non-Native Abundance**		**Relative Invasion**	
**Predictors**	**DF**	**F Value**	**P Value**	**F Value**	**P Value**	**F Value**	**P Value**	**F Value**	**P Value**	**F Value**	**P Value**
**Inoculation Treatment**	3	8.62	0.0003	3.45	0.0298	7.07	0.0011	5.25	0.0053	7.6	0.0007
**Block**	10	1.58	0.165	6.57	<0.0001	4.06	0.0016	2.12	0.0569	1.61	0.1559
**Contrasts**											
Inoculated vs. Non-Inoculated	1	15	0.0006	0.16	0.6958	12.37	0.0015	9.53	0.0045	15.13	0.0006
Having Native AM Fungi vs. Not	1	15.08	0.0006	3.89	0.0585	0.03	0.8666	1.74	0.1972	0.2	0.6568
Whole Soil vs. Whole Soil+AM Fungi	1	8.95	0.0057	10.19	0.0035	6.09	0.0199	0.31	0.5849	2.3	0.1406
Differences Among Living	2	5.43	0.0102	5.1	0.0129	4.41	0.0216	3.11	0.0605	3.83	0.0339

Table S3B. Harvest 1		**Early Successional**		**Late Successional**		**Native Forbs**		**Native Grasses**		**Native Legumes**	
**Predictors**	**DF**	**F Value**	**P Value**	**F Value**	**P Value**	**F Value**	**P Value**	**F Value**	**P Value**	**F Value**	**P Value**
**Inoculation Treatment**	3	4.65	0.0093	2.78	0.0597	7.31	0.0009	2.89	0.0528	1.43	0.2548
**Block**	10	3.96	0.0019	1.68	0.1342	3.61	0.0035	4.32	0.0011	2.73	0.0175
**Contrasts**											
Inoculated vs. Non-Inoculated	1	5.2	0.0304	8.06	0.0083	11.26	0.0023	0.62	0.4385	1.29	0.265
Having Native AM Fungi vs. Not	1	0.85	0.3648	2.53	0.1227	0.25	0.6207	1.72	0.2001	3.36	0.0777
Whole Soil vs. Whole Soil+AM Fungi	1	7.05	0.0129	0	0.9445	8.69	0.0064	4.98	0.0339	2.98	0.0951
Differences Among Living	2	4.38	0.0222	0.14	0.8725	5.34	0.0109	4.03	0.0289	1.5	0.2408

Table S4. The main effect of inoculation (bold) and contrasts results from our PROC GLM model in SAS on total abundance and by nativeness and successional stage (A) and for plant community diversity and by functional group (B) for the second harvest.

Table S4A. Harvest 2		**Total Diversity**		**Total Abundance**		**Native Abundance**		**Non-Native Abundance**		**Relative Invasion**	
**Predictors**	**DF**	**F Value**	**P Value**	**F Value**	**P Value**	**F Value**	**P Value**	**F Value**	**P Value**	**F Value**	**P Value**
**Inoculation Treatment**	3	17.86	<0.0001	2.01	0.134	15.04	<0.0001	18.28	<0.0001	18.8	<0.0001
**Block**	10	2.03	0.0662	2.06	0.0613	0.99	0.4725	1.86	0.0923	0.75	0.6713
**Contrasts**											
Inoculated vs. Non-Inoculated	1	44.59	<0.0001	0.41	0.5261	36.99	<0.0001	39.49	<0.0001	43.41	<0.0001
Having Native AM Fungi vs. Not	1	39.02	<0.0001	1.59	0.2176	32.99	<0.0001	17.98	0.0002	36.38	<0.0001
Whole Soil vs. Whole Soil+AM Fungi	1	8.2	0.0076	1.19	0.2843	7.66	0.0096	6.62	0.0153	12.53	0.0013
Differences Among Living	2	4.5	0.0195	2.81	0.0763	4.06	0.0276	7.68	0.002	6.49	0.0045

Table S4B. Harvest 2		**Early Successional**		**Late Successional**		**Native Forbs**		**Native Grasses**		**Native Legumes**	
**Predictors**	**DF**	**F Value**	**P Value**	**F Value**	**P Value**	**F Value**	**P Value**	**F Value**	**P Value**	**F Value**	**P Value**
**Inoculation Treatment**	3	8.04	0.0004	1.59	0.2128	5.15	0.0054	5.57	0.0037	14.43	<0.0001
**Block**	10	0.89	0.5512	0.86	0.5769	1.65	0.1407	1.88	0.0886	0.69	0.7239
**Contrasts**											
Inoculated vs. Non-Inoculated	1	21.31	<0.0001	1.66	0.2068	2.85	0.1017	14.7	0.0006	26.29	<0.0001
Having Native AM Fungi vs. Not	1	15.65	0.0004	3.63	0.0665	3.34	0.0776	2.57	0.1194	39.91	<0.0001
Whole Soil vs. Whole Soil+AM Fungi	1	2.74	0.1085	0.5	0.4836	6.34	0.0174	0	0.963	13.71	0.0009
Differences Among Living	2	1.41	0.2594	1.55	0.2287	6.3	0.0052	1	0.379	8.49	0.0012

Figure S1. Pots shown at harvest in July of 2017.

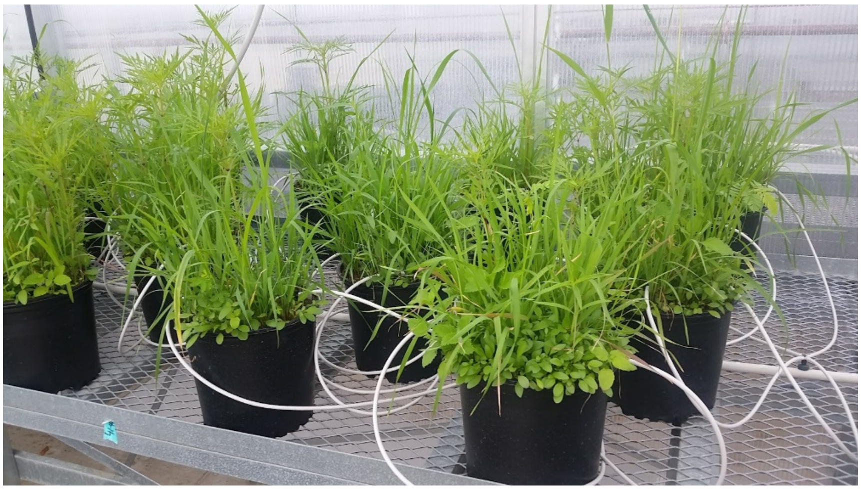

Figure S2. The effect of fungal composition native abundance (A), non-native abundance (B), early successional abundance (C), native forb abundance (D), native grass abundance (E), and total diversity (F) across harvests. Lines connect data from the first (black) or second (gray, double lined) harvest. Points represent the LS means of plant growth and error bars are standard error from the Proc GLM models. These panels include all metrics in which the main effect of inocula was consistent between harvests.

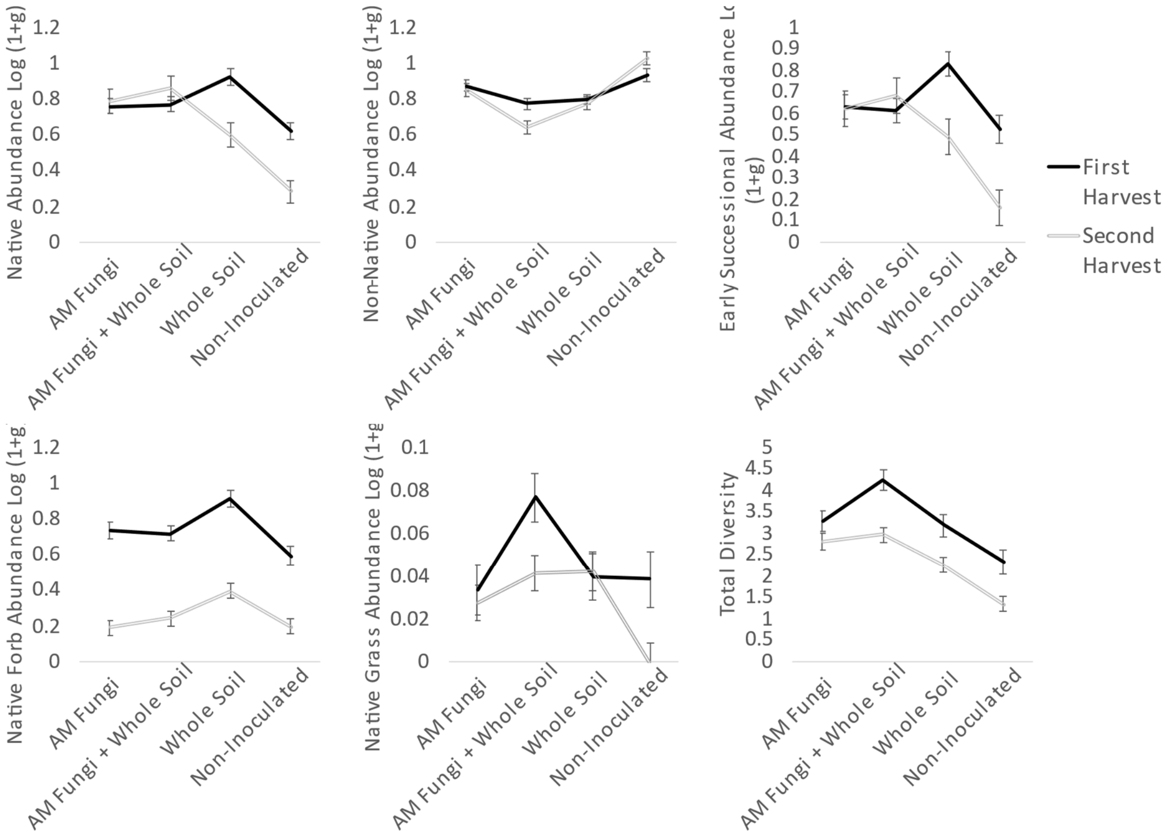

Figure S3. The effect of fungal composition on total mass (A), native legume abundance (B), the proportion of native plants (C), and late successional biomass (D). Lines connect data from the first (black) or second (gray, double lined) harvest. Points represent the LS means of plant growth and error bars are standard error. These panels include all metrics in which the main effect of inocula increased or decreased between harvests.

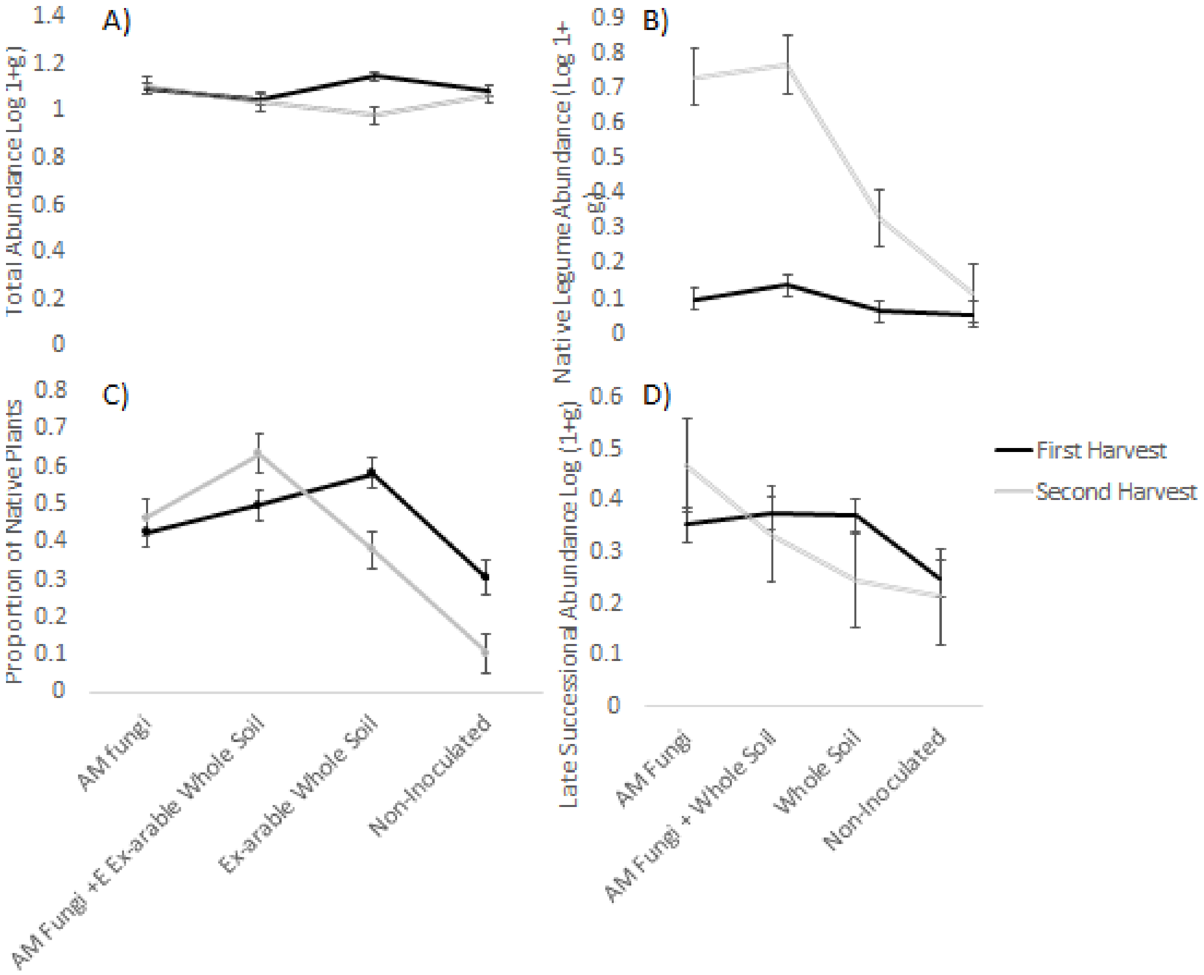

1 Freeman, C. Coefficients of Conservatism for Kansas Vascular Plants (2012) and Selected Life History Attributes. *Unpublished report.* (2014).
